# Efficacy of active hexose correlated compound on survival of patients with resectable/borderline resectable pancreatic cancer: a study protocol for a double-blind randomized phase II study

**DOI:** 10.1186/s13063-021-05934-x

**Published:** 2022-02-12

**Authors:** Daisuke Hashimoto, Sohei Satoi, Hideki Ishikawa, Yasuhiro Kodera, Keiko Kamei, Satoshi Hirano, Tsutomu Fujii, Kenichiro Uemura, Akihiko Tsuchida, Suguru Yamada, Tomohisa Yamamoto, Kiichi Hirota, Mitsugu Sekimoto

**Affiliations:** 1 Department of Surgery, Kansai Medical University, Osaka, Japan; 2 Division of Surgical Oncology, University of Colorado Anschutz Medical Campus, Aurora, CO USA; 3 Department of Molecular-Targeting Cancer Prevention, Graduate School of Medical Science, Kyoto Prefectural University of Medicine, Kyoto, Japan; 4 Department of Gastroenterological Surgery, Nagoya University Graduate School of Medicine, Nagoya, Japan; 5 Department of Surgery, Kindai University Faculty of Medicine, Osaka, Japan; 6 Department of Gastroenterological Surgery II, Hokkaido University Faculty of Medicine, Sapporo, Japan; 7 Department of Surgery and Science, Faculty of Medicine, Academic Assembly, University of Toyama, Toyama, Japan; 8 Department of Surgery, Institute of Biomedical and Health Sciences, Hiroshima University, Hiroshima, Japan; 9 Department of Gastrointestinal and Pediatric Surgery, Tokyo Medical University, Tokyo, Japan; 10 Department of Gastrointestinal and Pediatric Surgery, Nagoya Central Hospital, Nagoya, Japan; 11 Department of Human Stress Response Science, Institute of Biomedical Science, Kansai Medical University, Osaka, Japan

**Keywords:** Active hexose correlated compound, Functional food, Pancreatic cancer, Chemotherapy, Operation

## Abstract

**Background:**

The prognosis of pancreatic ductal adenocarcinoma remains very poor. One possible reason for the short survival of patients with this disease is malnutrition, which can be present at the initial diagnosis, and continue after pancreatectomy. Then, it is important to improve nutritional status and to decrease adverse events during neoadjuvant and adjuvant chemotherapy. Active hexose correlated compound (AHCC) is a standardized extract of cultured *Lentinula edodes* mycelia, and is considered a potent biological response modifier in the treatment of cancer. To evaluate the survival impact of AHCC on the patients with pancreatic ductal adenocarcinoma, we plan to perform this trial.

**Methods:**

This is a prospective multicenter phase II trial in patients with resectable/borderline resectable pancreatic ductal adenocarcinoma to investigate the efficacy of AHCC regarding survival. Patients will begin taking AHCC or placebo on the first day of neoadjuvant therapy. AHCC or placebo will be continued until 2 years after surgery. The primary endpoint will be 2-year disease-free survival. The secondary endpoints are the completion rate, dose intensity, and adverse event profile of preoperative chemotherapy; response rate to preoperative chemotherapy; rate of decrease in tumor marker (carbohydrate antigen 19-9, carcinoembryonic antigen) concentrations during preoperative chemotherapy; entry rate, completion rate, dose intensity, and adverse event profile of adjuvant chemotherapy; safety of the protocol therapy (adverse effect of AHCC); 2-year overall survival rate; and nutrition score before and after preoperative chemotherapy, and before and after adjuvant chemotherapy. We will enroll 230 patients, and the study involves eight leading Japanese institutions that are all high-volume centers in pancreatic surgery.

**Discussion:**

AHCC is expected to function as a supportive food in patients with pancreatic ductal adenocarcinoma, to reduce the proportion of severe adverse events related to neoadjuvant chemotherapy, and to increase the completion proportion of multimodal treatments, resulting in improved survival.

**Trial registration:**

The trial protocol has been registered in the protocol registration system at the Japan Registry of Clinical Trials (Trial ID: jRCTs051200029). At the time of the submission of this paper (October 2020), the protocol version is 2.0. The completion date is estimated to be November 2024.

## Administrative information

Note: the numbers in curly brackets in this protocol refer to SPIRIT checklist item numbers. The order of the items has been modified to group similar items (see http://www.equator-network.org/reporting-guidelines/spirit-2013-statement-defining-standard-protocol-items-for-clinical-trials/).

**Table:** 

Title {1}	A double-blind randomized phase II study of AHCC for patients with resectable/borderline resectable pancreatic cancer (Phase II study of AHCC for pancreatic cancer)
Trial registration {2a and 2b}.	The trial protocol has been registered in the protocol registration system at the Japan Registry of Clinical Trials (Trial ID: jRCTs051200029), Registered 25 June 2020 [1]. The jRCT is one of the WHO Primary Registries, which meet specific criteria for content, quality and validity, accessibility, unique identification, technical capacity and administration [2].
Protocol version {3}	The protocol version is 2.0 (August 20^th^, 2020).
Funding {4}	This work was supported by the Japan Society for the Promotion of Science KAKENHI Grant Number JP 20224644 (https://kaken.nii.ac.jp/grant/KAKENHI-PROJECT-20K11545/). The funding body had no control over the design of the study and collection, analysis, and interpretation of data and in writing the manuscript.
Author details {5a}	Daisuke Hashimoto,^1^ Sohei Satoi,^1^ Hideki Ishikawa,^2^ Yasuhiro Kodera,^3^ Keiko Kamei,^4^ Satoshi Hirano,^5^ Tsutomu Fujii,^6^ Kenichiro Uemura,^7^ Akihiko Tsuchida,^8^ Suguru Yamada,^9^ Tomohisa Yamamoto,^1^ Kiichi Hirota,^9^ Mitsugu Sekimoto.^1^ 1. Department of Surgery, Kansai Medical University, Osaka, Japan. 2. Department of Molecular-Targeting Cancer Prevention, Graduate School of Medical Science, Kyoto Prefectural University of Medicine, Kyoto, Japan. 3. Department of Gastroenterological Surgery, Nagoya University Graduate School of Medicine, Nagoya, Japan. 4. Department of Surgery, Kindai University Faculty of Medicine, Osaka, Japan. 5. Department of Gastroenterological Surgery II, Hokkaido University Faculty of Medicine, Sapporo, Japan. 6. Department of Surgery and Science, Faculty of Medicine, Academic Assembly, University of Toyama, Toyama, Japan. 7. Department of Surgery, Institute of Biomedical and Health Sciences, Hiroshima University, Hiroshima, Japan. 8. Department of Gastrointestinal and Pediatric Surgery, Tokyo Medical University, Tokyo, Japan. 9. Department of Gastrointestinal and Pediatric Surgery, Nagoya Central Hospital, Nagoya, Japan 10. Department of Human Stress Response Science, Institute of Biomedical Science, Kansai Medical University, Osaka, Japan.
Name and contact information for the trial sponsor {5b}	Sohei Satoi, MD, PhD, FACS Department of Surgery, Kansai Medical University 2-5-1 Shin-machi, Hirakata-city, Osaka, 573-1010 Japan Tel: +81-72-804-0101 Fax: +81-72-804-2578 E-mail: satoi@hirakata.kmu.ac.jp
Role of sponsor {5c}	This is an investigator initiated clinical trial. Therefore, the funder plays no role in the design of the study including data collection, analysis, and interpretation and manuscript writing.

## Introduction

### Background and rationale {6a}

Pancreatic ductal adenocarcinoma (PDAC) is now the fourth leading cause of cancer-related death in Japan. Recent progress in neoadjuvant and adjuvant chemotherapy has contributed to improved survival of PDAC patients after resection [3–6]. However, PDAC remains challenging to cure and the 5-year survival rate after PDAC resection is only 15–20% [7]. One possible reason for the short survival of patients with PDAC is malnutrition [8]. Malnutrition associated with the cancer burden may be present at the time of the initial diagnosis [9], and it is likely to continue after pancreatectomy, which is highly invasive and has a high morbidity rate [10–12]. Malnutrition can decrease the entry rate and completion rate of neoadjuvant and adjuvant chemotherapy for PDAC [13]. Thus, regarding the short- and long-term outcomes of PDAC patients, it is important to decrease adverse events and improve quality of life (QOL) and nutritional status during neoadjuvant and adjuvant chemotherapy.

Active hexose correlated compound (AHCC) is a standardized extract of cultured *Lentinula edodes* mycelia, produced by Amino Up Co., Ltd. (Sapporo, Japan) [14]. AHCC is a mixture of amino acids, minerals, lipids, and polysaccharides obtained from the fungi. It is a rich source of alpha-1,4-glucans, which are thought to enhance its biologic effects [14]. Previous studies have shown that AHCC has anti-inflammatory [15] and antioxidant effects [16], enhances resistance to bacterial [17] and viral infections [18], and exerts anticancer effects [15, 19–22]. AHCC has also been found to have synergistic effects with gemcitabine in pancreatic cancer cells [22]. In a murine model, AHCC increased the antitumor activity of cisplatin and mitigated the adverse effects of the chemotherapy agent [23]. AHCC also reportedly enhances natural killer cell activity, which may be associated with the incidence of cancer [24]. Thus, AHCC is considered a potent biological response modifier in the treatment of cancer [23, 25].

We previously reported in a retrospective study that AHCC intake improved the prognosis of postoperative hepatocellular carcinoma (HCC) patients [26]. Additionally, we indicated that AHCC intake effectively decreased the rate of adverse events and maintained the QOL of patients with unresectable PDAC during chemotherapy in a retrospective study [27]. AHCC has fewer adverse effect s[24] and can be considered safe in combination with chemotherapy on the basis of the latest evidence in the treatment of PDAC.

Various advantages of neoadjuvant therapy (NAT) for PDAC have recently been described, including early treatment of occult metastases, a reduced risk of intraoperative tumor seeding, and improved tolerance compared with postoperative therapy [28]. The PREP-02/JSAP05 study clearly revealed that NAT for PDAC was associated with significantly longer survival than was upfront surgery [4]. In addition, patients who underwent NAT showed significantly fewer pathological nodal metastases and a lower rate of hepatic recurrence than patients who underwent upfront surgery [4].

To our knowledge, no prospective studies of patients with PDAC have evaluated the efficacy of AHCC. To establish AHCC as a new standardized treatment in resectable (R)/borderline resectable (BR) PDAC patients, we plan to perform a multicenter double-blind randomized controlled phase II trial.

### Objectives {7}

AHCC is an inherent part of multidisciplinary therapy to prolong survival after NAT and surgery for R/BR-PDAC [26, 27]. This trial is a prospective multicenter double-blind randomized phase II study that is planned for patients with R/BR-PDAC. The aim of this study is to investigate the improvement in 2-year disease-free survival (DFS), beginning after the first day of initial therapy, in patients with PDAC who receive NAT with AHCC followed by surgery compared with patients who undergo NAT with placebo.

### Trial design {8}

**Fig. 1:**
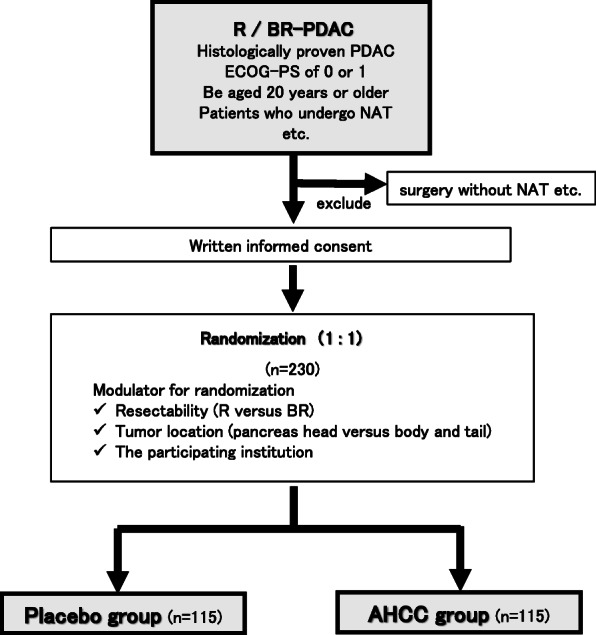
Trial flow diagram. AHCC, active hexose correlated compound; BR, borderline resectable; ECOG-PS, Eastern Cooperative Oncology Group performance status; NAT, neoadjuvant therapy; PDAC, pancreatic ductal adenocarcinoma; R, resectable

**Fig. 2:**
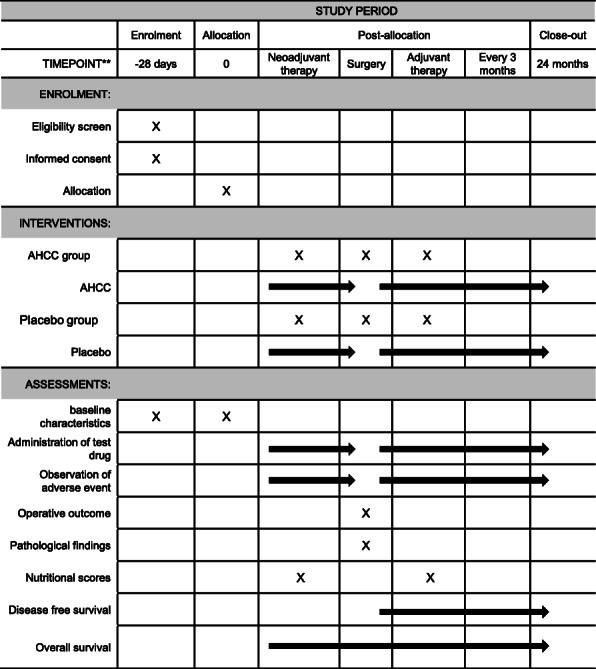
Summary of study assessments and procedures. AHCC, active hexose correlated compound

Patients with PDAC who undergo preoperative chemotherapy randomized in a 1:1 allocation ratio to either the AHCC group or the placebo group ratio (Fig. 1). The schedule of this trial is shown in Fig. 2. This study is designed to evaluate the superiority of AHCC therapy compared with placebo in terms of the 2-year DFS rate. Signs of PDAC recurrence will be monitored every 3 months for at least 2 years after surgery, using enhanced computed tomography (CT) or magnetic resonance image (MRI). The study period is expected to be 4 years, comprising 2 years for patient recruitment and 2 years for follow-up.

## Methods: Participants, interventions, and outcomes

### Study setting {9}

This trial will be conducted at Kansai Medical University Hospital and six Japanese high-volume centers, namely Hiroshima University, Hokkaido University Faculty of Medicine, Kindai University Faculty of Medicine, Nagoya University Graduate School of Medicine, Tokyo Medical University, and the University of Toyama.

### Eligibility criteria {10}

#### Inclusion criteria

The inclusion criteria for this trial are as follows:

1. Histologically-proven PDAC.
2. R and BR-PDAC defined according to the General Rules for the Study of Pancreatic Cancer (7th Edition, Japan Pancreas Society) [29].
3. Patients undergoing NAT.
4. Eastern Cooperative Oncology Group performance status of 0 or 1.
5. Adequate bone marrow, liver, and kidney function in measurements taken within 14 days before registration (leukocyte count ≥ 3000 cells per mm^3^ and ≤ 12,000 cells per mm^3^; neutrophil count ≥ 2000 cells per mm^3^; hemoglobin concentration ≥ 8.0 g/dL; platelet count ≥ 100,000 cells per mm^3^; total bilirubin ≤ 2.0 mg/dL (3.0 mg/dL for patients with biliary stents); aspartate aminotransferase and alanine aminotransferase concentrations ≤ 150 IU/L; serum creatinine concentrations ≤ 1.2 mg/dL; and creatinine clearance ≥ 50 mL/min).
6. Possibility of adequate oral intake.
7. Written informed consent.
8. Age ≥ 20 years, with no cognitive limitations.

#### Exclusion criteria

The exclusion criteria in this trial are as follows:

1. Unresectable pancreatic cancer owing to distant metastasis (e.g., distant lymph nodes, liver, peritoneal dissemination, lung, pleural dissemination, brain, and bone) according to the General Rules for the Study of Pancreatic Cancer (7th Edition) [29].
2. Local advanced unresectable pancreatic cancer according to the General Rules for the Study of Pancreatic Cancer (7th Edition) [29].
3. Previous treatment for pancreatic cancer before registration, such as with chemotherapy, radiation, or immune therapy.
4. Patients who undergo surgery without NAT (upfront surgery).
5. Serious drug allergy to the combination of tegafur, gimeracil, and oteracil potassium (S-1); gemcitabine; nab-paclitaxel; paclitaxel; oxaliplatin; irinotecan; leucovorin; or fluorouracil.
6. Active infectious disease (e.g., pyrexia ≥ 38 °C body temperature).
7. Other serious comorbidities (eg, heart failure, kidney failure, pulmonary failure, liver failure, or uncontrolled diabetes).
8. Gastrointestinal bleeding requiring repeat blood transfusions.
9. Inadequately controlled watery diarrhea.
10. Complicating psychiatric disorder or psychological symptoms.
11. Active treatment at multiple primary cancers.
12. Pregnant, breastfeeding, childbearing potential, or willingness to bear children.
13. Receiving flucytosine, phenytoin, or warfarin potassium.
14. Unable to provide written informed consent.

### Who will take informed consent? {26a}

Attending researchers of each institution will take written informed consent for the trial from each patient before enrollment. The treatment protocol, benefits, risks, and data management of this study will be clarified in detail for the patients.

### Additional consent provisions for collection and use of participant data and biological specimens {26b}

As an additional study, the stool of the participants will be analyzed to make clear the change of microbiome after chemotherapy and the influence of AHCC. For this additional study, another informed consent will be obtained from the participants again.

## Interventions

### Explanation for the choice of comparators {6b}

To make clear the survival impact of AHCC, participants of the control group of this trial will take placebo.

### Intervention description {11a}

We previously performed a double-blind, placebo-controlled trial and evaluated the effects of AHCC intake on immune responses in healthy volunteers [24]. In that study, subjects were randomized to receive placebo or AHCC at 3.0 g/day for 4 weeks. None of the subjects showed any adverse effects and none withdrew during the study period. According to our previous study, patients enrolled in this study will receive AHCC at 3.0 g/day.

The AHCC and placebo used in this study will be manufactured exclusively by Amino Up Co., Ltd. The AHCC and placebo will be delivered as a powder and stored at room temperature. The placebo will contain dextrin (40%), malt extract (40%), and hardened oil (20%).

**Fig. 3:**
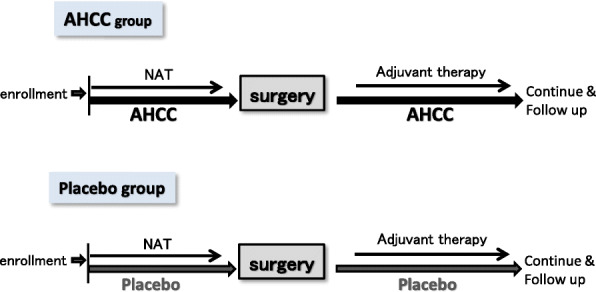
Trial protocol. AHCC, active hexose correlated compound; NAT, neoadjuvant therapy

The protocol of this trial is described in Fig. 3. Patients will begin taking AHCC or placebo at 3.0 g/day (1.0 g × 3 times/day), on the first day of NAT. AHCC or placebo will be continued until the day before surgery and will be restarted when patients start eating again after surgery. AHCC and placebo will be continued during and after adjuvant chemotherapy until 2 years after surgery. The regimen for NAT and adjuvant chemotherapy will depend on the treatment strategy at each participating institution. The PREP-02/JSAP05 study recruited patients with R-PDAC and BR-PDAC, and most (80%) of the registered patients had R-PDAC [4]. With reference to that study, we expect that most of the patients included in our trial will have R-PDAC. Gemcitabine plus S-1 will be used as NAT for many patients in this trial because this is the standard NAT regimen for R-PDAC in the Japanese guideline [4, 30]. In addition, several regimens will be considered for NAT in both groups, such as gemcitabine plus nab-paclitaxel [31]; oxaliplatin, irinotecan, fluorouracil, and leucovorin (FOLFIRINOX) [32]; and S-1, oxaliplatin, and irinotecan (SOXIRI) [33].

### Criteria for discontinuing or modifying allocated interventions {11b}

When participating patients want to leave the study, they can do so at any time for any reason without any consequences. When adverse events associated with AHCC or placebo at grade 3 or more of the Common Terminology Criteria for Adverse Events (CTCAE) are observed [34], AHCC or placebo will be decreased to 2.0 g/day. Those can be decreased to 1.0 g/day in the next step, and be terminated finally.

### Strategies to improve adherence to interventions {11c}

Basically, the adherence to this protocol will be high because this trial is double-blinded. Medication adherence rate will be monitored using a medication adherence diary.

### Relevant concomitant care permitted or prohibited during the trial {11d}

During the protocol therapy, any anticancer therapy except for NAT and adjuvant therapy, such as immunotherapy, endocrine therapy, and thermotherapy will be not permitted.

### Provisions for post-trial care {30}

After finishing the follow-up period, the patient is followed in terms of routine surveillance and treated if necessary.

### Outcomes {12}

The primary endpoint will be the 2-year disease-free survival (DFS) rate. It will begin from the first day of the protocol therapy, and end at the day of confirmed postoperative recurrence. The DFS will be measured in months. For patients who do not undergo surgical resection because of disease progression (i.e., progression during NAT, unresectable or metastatic tumors found during surgery), DFS will be defined from the first day of the protocol therapy to the date of disease progression. The secondary endpoints will be the completion rate and dose intensity of preoperative chemotherapy; response rate of preoperative chemotherapy; decreasing tumor marker (carbohydrate antigen 19-9, carcinoembryonic antigen, duke pancreatic monoclonal antigen type 2) concentrations during preoperative chemotherapy; rate of patients undergoing postoperative adjuvant therapy (entry rate, completion rate); dose intensity of postoperative adjuvant therapy; safety of the protocol therapy (adverse effects); 2-year overall survival (OS) rate; and nutritional scores, such as the neutrophil to lymphocyte ratio (NLR) [35, 36], prognostic nutrition index (PNI) [37, 38], C-reactive protein to albumin ratio [39], modified Glasgow prognostic score (mGPS) [40], and platelet to lymphocyte ratio (PLR) [41]. Nutritional scores will be evaluated before and after preoperative chemotherapy, and before and after postoperative adjuvant chemotherapy. Adverse effects during NAT and adjuvant chemotherapy will be scored based on the CTCAE, Version 4.0 [34]. Postoperative pancreatic fistula (POPF) [42], delayed gastric emptying (DGE) [43], and postpancreatectomy hemorrhage (PPH) [44] will be graded based on the International Study Group of Pancreatic Surgery. Postoperative complications other than POPF, DGE, and PPH will be graded using the Clavien–Dindo classification system [45].

### Participant timeline {13}

Figure 3 also shows the timeline for the participating patients. After enrollment, the patients will start NAT and AHCC or placebo within 3 weeks. Post-surgery, the patients in this study will be followed every 3 months for at least 2 years and enhanced CT or MRI will be performed every 3 months to evaluate postoperative recurrence and metastases.

### Sample size {14}

A recent prospective trial of R/BR-PDAC, the Prep-02/JSAP05 study, demonstrated a significant survival benefit of NAT followed by surgery over upfront surgery for PDAC [46]. The study showed that the 2-year DFS rates were 36.1% for patients with PDAC treated with NAT followed by surgery. Therefore, in our planned trial we set the 2-year DFS of the placebo group at 36.1%.

We previously indicated that AHCC intake significantly improved the prognosis of postoperative HCC patients [26]. In the study, the 2-year DFS rate was 72.0% in the AHCC group and 50.0% in the control group, which did not receive AHCC. On the basis of this ratio, we set the 2-year DFS in the AHCC group in our planned trial at 55.5%. To confirm the superiority of the 2-year DFS of the AHCC group compared with the placebo group using the *χ*^2^ test and a significance level of 0.05, 216 patients (108 patients in each group) will be necessary [47]. Anticipating a dropout rate of 6% for each group, 230 patients (115 patients in each group) will be recruited in this trial (Fig. 1).

### Recruitment {15}

To collect adequate participant enrolment and achieve the target sample size within the study period, 8 high-volume centers in Japan participate in this trial. All eligible patients visiting a participating facility will be screened. Participating physicians will explain the trial to patients using a flyer, and patients who express interest will be given further details using a consent statement.

## Assignment of interventions: allocation

### Sequence generation {16a}

Computer-generated randomization will be used for generating the allocation sequence. The participating patients will be randomized in a 1:1 allocation ratio to either the AHCC group or the placebo group, with a random block size (Fig. 1). To minimize background bias between the two groups, this study will be stratified for resectability (R versus BR), tumor location (pancreas head versus body and tail), and the participating institution.

### Concealment mechanism {16b}

The participating patients will be randomized using the computer randomization module. Central randomization will be done for this trial to ensure allocation concealment.

### Implementation {16c}

After signing the informed consent forms and assessment for eligibility at registration, the researchers of the participating hospitals will use the computer randomization module to allocate the patient. After filling the stratification factors described as above, the researchers will click the randomize button which will provide the allocation information. This information will be recorded in the database and cannot be changed in future.

## Assignment of interventions: Blinding

### Who will be blinded {17a}

This trial is a double-blind randomized, and the researchers and surgeons of the participating hospitals, the participating patients, and analysts who analyze the data will be blinded to the therapy that patients will receive. Only the data manager can tell whether the patient belongs to the AHCC group or the placebo group. The data manager and the researchers and surgeons of the participating hospitals will use a common patient number to manage participating cases. The patient number does not include information on whether the patient is in the AHCC or placebo group. The AHCC and placebo will have the same appearance, smell, and flavor. The study drug given to the researchers and surgeons of the participating hospitals and the patients by the data manager does not specify whether it is an AHCC or placebo.

### Procedure for unblinding if needed {17b}

We do not anticipate any requirement for unblinding but if required, the Trial Manager/Surgeon/GP, Data Coordinator, Implementation Support Facilitators will have access to group allocations and any unblinding will be reported.

## Data collection and management

### Plans for assessment and collection of outcomes {18a}

Patients will use medication adherence diary to be monitored adherence rate and to answer questionnaires. Laboratory tests and imaging tests such as CT and MRI are performed in each institution by the researchers and surgeons of the participating hospitals. Data from electronic patient records, including information of recurrence and survival, will be collected with a case report form (CRF) by the researchers in each hospital. The CRFs and medication adherence diaries will be sent to an independent data center (Medical Research Support, Ltd., Osaka, Japan). The data manager in the data center will receive those and entered source data collected from those into a password-secure electronic database. The CRFs and database will be routinely checked for accuracy by the data manager during the data collection period. The final database will be locked after the resolution of all queries. All paper CRFs will be filed and kept in lockable cabinets in the data center accessible only to study personnel. At the conclusion of the study, the files will be stored for a period of 5 years after study completion. The data center, an independent statistician, and an independent data and safety monitoring committee have access to the data folder of this trial.

### Plans to promote participant retention and complete follow-up {18b}

The patients will be explained about their schedule including visiting the hospital and the importance of completion of the follow-up at the registration. The patients can stop at any time whenever they decide during the protocol or the follow-up period without giving a reason to discontinue. Throughout the follow-up period, the researchers will encourage the patients for completion of their follow-up.

### Data management {19}

The data and safety monitoring committee will monitor the safety of this trial every 6 months by qualitative analyses of feasibility, accrual rate, adverse events, and dropouts from the trial. The data center will collect data securely via a paper case report form, which will be stored and managed by the committee. After patients provide signed informed consent, baseline assessments will be conducted before randomization. Missing data will be stored until received or confirmed as not available or until this trial reaches analysis.

### Confidentiality {27}

Patients’ data will be managed using anonymized registration numbers. Each participating institution will store the correspondence table of the anonymization codes, and patients’ names and identifying consent forms, in a restricted-access lockable document storage unit.

### Plans for collection, laboratory evaluation, and storage of biological specimens for genetic or molecular analysis in this trial/future use {33}

As an additional study, the stool of the participants will be collected for four or five times: before NAT, before surgery, before adjuvant chemotherapy, after adjuvant chemotherapy, and at a time of recurrence if available.

## Statistical methods

### Statistical methods for primary and secondary outcomes {20a}

The intention-to-treat analysis set will be the full analysis set, defined as all randomized patients. The per-protocol analysis will involve the patients who undergo surgical resection of PDAC. The primary endpoint will be compared using a Cox proportional hazards model with a two-sided alpha of 0.05 stratified by resectability (R versus BR), tumor location (pancreas head versus body and tail), and the participating institution.

Survival analysis will be conducted using Kaplan–Meier survival curves in the two randomized groups. For the secondary endpoints, categorical outcomes will be summarized using frequency and percentage in each arm and will be compared by Fisher’s exact test. Continuous outcomes will be described as median and range for each arm and will be compared using Wilcoxon’s test.

DFS is defined as the time from surgery to the time of finding any recurrence or metastasis, or until death. OS is defined as the time from surgery to the time of the last follow-up, or death.

### Interim analyses {21b}

No interim analyses are planned in this trial. However, when half of the sample (*n*=115) has been recruited intermediate-term results will be examined by the data and safety monitoring committee to confirm the safety of this trial. The data and safety monitoring committee will check incidence of adverse events of each group and will make a recommendation to the principal investigator (Sohei Satoi) as to whether continuation of the study is appropriate. Additionally, the principal investigator will be able to terminate the trial in consultation with the independent statistician.

### Methods for additional analyses (e.g., subgroup analyses) {20b}

There are no subgroup analyses planned.

### Methods in analysis to handle protocol non-adherence and any statistical methods to handle missing data {20c}

Missing data should be reduced to a minimum. The patient will be censored at the date of the last follow-up unless the patient is documented to have an event. The researchers will encourage the patients to ensure adherence to follow-up. The patients will make an appointment for a next medical examination at every hospital visit.

### Plans to give access to the full protocol, participant-level data, and statistical code {31c}

The protocol is available at the protocol registration system at the Japan Registry of Clinical Trials (Trial ID: jRCTs051200029) [1]. There is no plan to publish patient-level data; however, we will consider after the completion of reporting of the outcomes.

## Oversight and monitoring

### Composition of the coordinating center and trial steering committee {5d}

All researchers of the participating hospitals will be a part of the trial management committee. Study coordination will be performed by the Department of Surgery, Kansai Medical University. Data acquisition will be performed by the data center. Statistical analysis will be performed by the independent statistician. In addition, we constitute the data and safe monitoring committee who will provide an independent assessment of the safety of the trial.

### Composition of the data monitoring committee, its role and reporting structure {21a}

The independent data safety and monitoring committee is constituted which will provide an assessment of the patient safety and recommendation to the principle investigator and the researchers about the continuation of this trial. The assessment of the data safety and monitoring committee will be communicated to the Wakayama Medical University Certified Review Board (CRB) which certified this trial. The data safety and monitoring committee consists of three members who did not belong to the participating hospitals.

### Adverse event reporting and harms {22}

All adverse events observed during the study period will be evaluated based on the Common Terminology Criteria for Adverse Events (CTCAE) criteria v5.0, and recorded in a CRF to report it [34]. The assessment of the severity of adverse events will be made by the researcher responsible for the care of the patient. The adverse events reporting CRF will be sent to the data manager and the data and safety monitoring committee. Severe adverse events (SAE) are defined as those that are requiring hospitalization, life-threatening, or result in death. SAEs will be reported to the principal investigator, the data manager, and the data and safety monitoring committee within 48 working hours of the study staff first becoming aware. Other adverse events will be collected and reported according to a planned schedule (Fig. 2).

### Frequency and plans for auditing trial conduct {23}

Auditing will be performed after completion of enrollment of 230 cases by a study monitor which is independent from the study group. Any protocol deviation from the protocol should be notified to the CRB and the study group and discussed in the trial management meetings. Additional audits and monitoring would be done as appropriate.

### Plans for communicating important protocol amendments to relevant parties (e.g., trial participants, ethical committees) {25}

Changes to the protocol will be made by the Trial Management Committee, as necessary. Approval of the changes by the CRB is required prior to their implementation. An updated protocol will be shared through email as well as during regular trial management committee meetings and stored and published at the Japan Registry of Clinical Trials (Trial ID: jRCTs051200029) [1].

### Dissemination plans {31a}

The results of this trial will be disclosed completely at an international conference and submitted to a peer-reviewed journal. Both positive and negative results will be reported.

## Discussion

We plan to conduct a double-blind randomized phase II study of AHCC for patients with R/BR-PDAC. To our knowledge, this trial is the first prospective trial to evaluate the efficacy of AHCC as a functional food. We will enroll patients with R/BR-PDAC and evaluate DFS.

The prognosis of PDAC remains very poor because of delayed disease detection and the limited effectiveness of systemic therapies. Recent improvements in multimodal treatment, such as more active chemotherapy regimens, targeted therapies, and immunotherapy approaches, have substantially contributed to the prolonged OS, greater local control, and longer DFS for patients with PDAC [48]. Moreover, a multidisciplinary approach in the neoadjuvant setting is considered important for patients with R-PDAC.

AHCC is expected to reduce the proportion of severe adverse events related to neoadjuvant and adjuvant chemotherapy and to increase the completion proportion of multimodal treatments, resulting in improved DFS and OS. AHCC functions as a supportive food during chemotherapy in patients with PDAC who have malnutrition owing to the tumor burden and who undergo high-toxicity chemotherapy.

This trial involves several limitations that should be considered. First, the NAT and adjuvant therapy regimens in this trial are not defined. Most of the patients who will be enrolled are expected to have R-PDAC. Because gemcitabine plus S-1 is the standard NAT regimen in Japan [46], this regimen will be used for many cases in this trial. Several regimens will be used for BR-PDAC, such as gemcitabine plus nab-paclitaxel [31], FOLFIRINOX [32], and SOXIRI [33]. However, almost all participating patients will receive S-1 as adjuvant therapy because this is the standard therapy indicated in the Japanese guideline [30]. The NAT and adjuvant therapy regimens will be limited or stratified in a future trial. Second, because no previous study has evaluated the long-term survival of patients with resected PDAC treated with AHCC, we set the 2-year DFS in the AHCC group according to the results of a previous study of HCC [26].

If this trial shows that AHCC treatment improves survival and the nutritional status of patients with R/BR-PDAC who undergo NAT followed by surgery, we expect AHCC to become a standard treatment for PDAC, and will plan an international multicenter randomized controlled phase III trial involving high-volume centers worldwide.

## Trial status

This enrolment of participants in this trial started in June 2020. At the time of the submission of this paper (October 2020), the protocol version is 2.0 [1]. The completion date is estimated to be November 2024.

## Abbreviations

AHCC: Active hexose correlated compound
BR: Borderline resectable
CRB: Certified Review Board
CRF: Case report form
CT: Computed tomography
CTCAE: Common Terminology Criteria for Adverse Events
DFS: Disease-free survival
DGE: Delayed gastric emptying
FOLFIRINOX: Oxaliplatin, irinotecan, fluorouracil, and leucovorin
HCC: Hepatocellular carcinoma
MRI: Magnetic resonance image
NAT: Neoadjuvant therapy
OS: Overall survival
PDAC: Pancreatic ductal adenocarcinoma
POPF: Postoperative pancreatic fistula
PPH: Postpancreatectomy hemorrhage
QOL: Quality of life
R: Resectable
S-1: Tegafur, gimeracil, and oteracil potassium
SOXIRI: S-1, oxaliplatin, and irinotecan
